# Plant Polyphenols as Chemopreventive Agents for Lung Cancer

**DOI:** 10.3390/ijms17081352

**Published:** 2016-08-19

**Authors:** Madumani Amararathna, Michael R. Johnston, H. P. Vasantha Rupasinghe

**Affiliations:** 1 Department of Environmental Sciences, Faculty of Agriculture, Dalhousie University, P.O. Box 550, Truro, NS B2N 5E3, Canada; madu.ama@dal.ca; 2 Department of Surgery, Dalhousie University, Halifax, NS B3H 4R2, Canada; mrj2@mac.com; 3 Department of Pathology, Faculty of Medicine, Dalhousie University, P.O. Box 15000, Halifax, NS B3H 4R2, Canada

**Keywords:** polyphenols, lung cancer, carcinogenesis, chemoprevention, diet, fruits and vegetables

## Abstract

Lung cancer may be prevented by a diet rich in fruits and vegetables as they are enriched with dietary antioxidant polyphenols, such as flavonoids, proanthocyanidins, lignans, stilbenes, and phenolic acids. Dietary polyphenols exert a wide range of beneficial biological functions beyond their antioxidative properties and are involved in regulation of cell survival pathways leading to anticarcinogenic and antimutagenic functions. There are sufficient evidence from in vitro, in vivo, and epidemiological studies to suggest that the dietary intervention of polyphenols in cancer prevention, including the chemopreventive ability of dietary polyphenols, act against lung carcinogens. Cohort and epidemiological studies in selected risk populations have evaluated clinical effects of polyphenols. Polyphenols have demonstrated three major actions: antioxidative activity, regulation of phase I and II enzymes, and regulation of cell survival pathways against lung carcinogenesis. They have also shown an inverse association of lung cancer occurrences among high risk populations who consumed considerable amounts of fruits and vegetables in their daily diet. In in vitro cell culture experimental models, polyphenols bind with electrophilic metabolites from carcinogens, inactivate cellular oxygen radicals, prevent membrane lipid peroxidation and DNA oxidative damage, and adduct formation. Further, polyphenols enhance the detoxifying enzymes such as the phase II enzymes, glutathione transferases and glucuronosyl transferases.

## 1. Introduction

Worldwide cancer statistics document the devastating effects of lung cancer. Among all types of cancer, lung cancer has caused almost one fifth of cancer deaths (19.4%). Incidence and mortality rates of lung cancer were estimated to be higher in less developed regions than developed regions. Lung cancer is the most common cancer in men worldwide [1]. In addition to the effect from life loss, the global economic impact (disability, treatment cost, palliative care) of lung cancer alone was $188 billion in 2008 [2]. Thus, lung cancer has become a serious concern throughout the world. 

Lung cancer is a multi-step process of accumulating genetic and epigenetic alterations caused by chronic exposure to carcinogens, such as smoking, domestic radon, fossil fuel burning, automobile exhaust fumes and pesticide inhalation. As per the World Health Organization (WHO), at least one-third of all cancer cases are preventable through dietary modifications and lifestyle changes. Eliminating risk factors, such as cigarette smoke and polluted environments, and adopting safety measures at work are a few actions for lung cancer prevention suggested by the WHO [3,4,5]. Many scientists have observed an inverse association between the consumption of fresh fruits and vegetables and the risk of lung cancer [6,7]. Therefore, in this review, a polyphenols-rich dietary preventive strategy is discussed in relation to lung cancer.

## 2. Nature of Polyphenols and Their Classification

**Figure 1:**
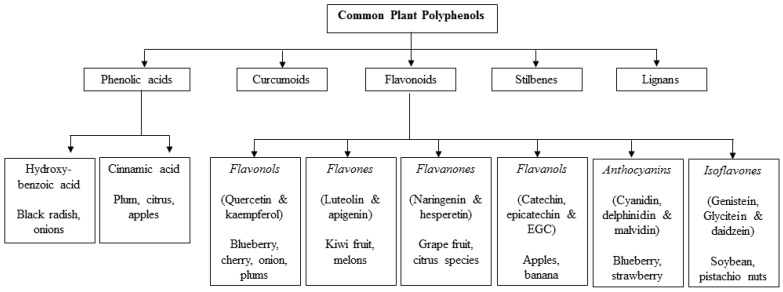
Classification of polyphenols, examples and dietary sources [11,12,13,14,15,16,17]. EGC—epigallocatechin.

Plant-based biologically active compounds have gained interest due to their disease prevention capability. Polyphenols present in plant-based foods, such as fruits and vegetables, reduce the risk of a variety of cancers. Polyphenols are the largest group of over 40,000 secondary plant metabolites that provide plants with chemical defense mechanisms against pathogens and environmental stress, as well as establishing many plant-ecological interactions [8]. Plant-based diets consumed around the world are rich in polyphenols such as phenolic acids, flavonoids, stilbenes and lignans. There are a wide variety of polyphenols identified so far and they contain at least one aromatic ring with one or more hydroxyl groups [9,10]. Among them, flavonoids have a basic structure, consisting of two aromatic rings (A and B rings) linked by three carbon atoms that are confined in an oxygenated heterocycle ring (C ring). Based on their differences in the C ring, flavonoids are further classified as flavonols, flavones, catechins/condensed tannins, anthocyanidins, and isoflavones. Other polyphenols differ from each other by the presence of hydroxyl, methoxyl, and/or glycosyl groups in their structures. (Figure 1).

## 3. Characteristics of Polyphenols in Cancer Prevention

Hydroxyl groups present in polyphenols (phenol ring) have the capability to donate hydrogen molecules for free radicals and convert free radicals into chemically stable less reactive molecules. Some polyphenols, such as epigallocatechin-3-gallate (EGCG), epicatechin gallate (ECG), and epicatechin (EC), can form metal chelates, thus preventing the formation of metal-catalyzed free radicals through Fenton reactions. Oxidative damage of lipids, proteins, and nucleic acids is prevented as a result of lower accumulation of free radicals and oxidant species in the biological system [18]. Moreover, hydroxyl groups are able to make hydrogen bonds with biological membranes and alter regulation of membrane-bound enzymes and receptors. Quercetin has been identified as a potent antagonist for the aryl hydrocarbon receptor (AhR) which regulates expression of cytochrome P450 (CYP) enzymes (capable of activating procarcinogens) and a transcription factor that can be activated by polycyclic aromatic hydrocarbons (PAHs) implicated in lung carcinogenicity [19,20,21]. Phenolic hydroxyl groups donate hydrogen groups and react with reactive oxygen and reactive nitrogen species [22], which break the cycle of new radical generation and stabilize the radicals. The presence of a hydrophobic benzoic ring and the capability for hydrogen bonding help polyphenols to interact with proteins present in cells. Hence, they suppress the activity of radical generating enzymes such as cytochrome 450 isoforms, lipoxygenases and cyclooxygenase and reduce intracellular oxidative stress [23,24].

Phase II enzymes generally detoxify endogenous and xenobiotic electrophilics (from chemical carcinogens) through glucuronidation, sulfation, methylation, acetylation, glutathione, and amino acid conjugation. The resulting hydrophilic compounds are easily excreted via bile or urine [25]. For example, isoflavone genistein, which is rich in soybean, has exhibited the ability of inducing phase II detoxifying and antioxidant enzymes in cell culture models through activating the extracellular signal-regulated protein kinase 1/2 (ERK1/2) and protein kinase C (PKC) signaling pathway, and increasing nuclear factor E2-related protein 2 (Nrf2), which interact with antioxidant response element (ARE). Most genes encoding phase II enzymes contain an ARE sequence in their promoter regions [26]. Nrf2 protects the cells against the formation of DNA adducts and/or gene mutations from benzo[a]pyrene (BaP). Diesel exhaust fumes and up-regulation of ARE-driven genes, by activated Nrf2, enables the cells to protect against increased concentration of electrophiles, free radicals, and reactive oxygen, nitrogen, and sulphur species [27,28,29,30].

Consumption of fruits and vegetables has given significant protection in 24 of 25 studies in lung cancer [31]. Quercetin from onions and apples was found to be inversely associated with lung cancer risk, especially against squamous cell carcinoma [32,33]. A population-based case control study (1061 cases and 1425 controls) conducted by Christensen and colleagues (2012) has shown that lower dietary flavonoid intake can increase the lung cancer risk. In addition, they found an inverse association between intake of flavone and flavanone-rich diets and squamous cell carcinoma incidences, but no association was found with adenocarcinoma incidences [34]. Many in vitro studies have justified the role of polyphenols in lung cancer control (Table 1).

## 4. Environmental and Occupation Lung Carcinogens

Lung cancer is not a sudden event; it arises from long-term accumulation of genetic and epigenetic modifications occurring in lung cells. It is a heterogeneous disease condition [42]. Lung cancer is classified pathohistologically into two broad categories: small-cell lung cancer (SCLC) (about 15%) and non-small cell lung cancer (NSCLC) (about 85%) [43]. In all SCLC tumors, deletion of 3p(14–23) in the region containing the tumour suppressor gene *FHIT* (a member of the histidine triad gene family) is seen. Tyrosine kinase signaling genes, including *KRAS* and *EGFR*, are rarely mutated [44]. The loss of tumour-suppressor gene retinoblastoma (*RB1*) and the mutation of tumour suppressor gene *TP53* are more common in SCLC patients than among NSCLC patients. Loss of the activity of tumour suppressor genes at the early stage of SCLC development can decrease apoptosis, induce cell proliferation and increase the survival of cancer cells [45].

NSCLC is the leading cause of cancer deaths worldwide with a 14% five-year survival across all stages of the disease [46]. NSCLC is classified into three major sub-groups: squamous cell carcinomas (SCC), adenocarcinomas (ADC), and large cell carcinomas (LCC) and into several minor sub-groups: adenosquamous and sarcomatoid carcinomas [47]. SCC are located centrally while ADC and LCC are usually found in the peripheral lung tissues. In lung cancer histology, SCC consists of keratinized cells tightly attached by intracellular cell junctions, but ADC shows glandular formation and/or mucin production whereas LCC have undifferentiated characteristics [48]. Early stage lung cancer can be treated with curative intent by surgery or, in some cases, with radiotherapy. However, most lung cancers are diagnosed at the later stage of disease with extensive local-regional involvement and systemic metastases. These patients have a poor prognosis and are treated mostly with systemic chemotherapy and palliative radiotherapy [49].

The International Agency for Research on Cancer (IARC) has classified lung carcinogenic agents into five broad groups:

1. Group 1: Carcinogenic to human.
2. Group 2A: Probably carcinogenic to human.
3. Group 2B: Possibly carcinogenic to human.
4. Group 3: Not classifiable as it’s carcinogenic to human.
5. Group 4: Probably not carcinogenic to humans.

Carcinogens which have demonstrated sufficient evidence of lung carcinogenesis have been classified as group I lung carcinogens (Table 2).

Only 1% of lung cancers originate from the inheritance of a germ line mutation. Most are associated with somatic mutations due to environmental or occupational exposures and lifestyle factors. These mutations may occur in oncogenes, tumor suppressor genes, cell cycle control genes, DNA repair genes, apoptosis regulator genes, and telomerase associate genes [75].

**Figure 2:**
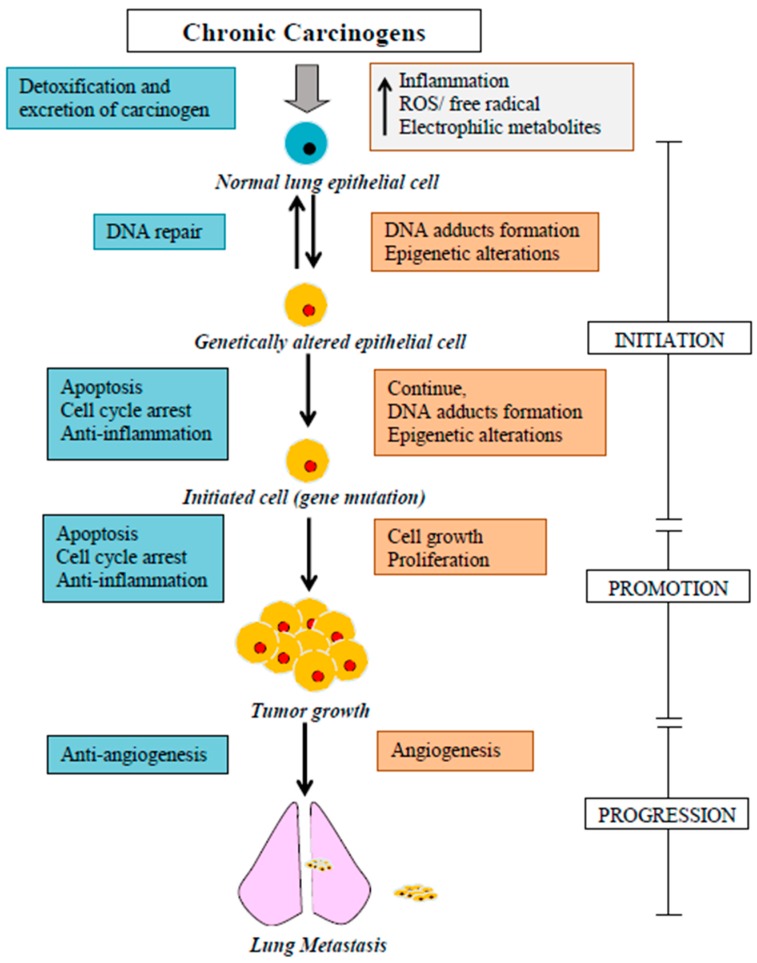
Stages of developing lung cancer, in summary (Modified from Rupasinghe et al., 2014 [8]).

Lung carcinogenesis is a complex cascade of molecular and cellular alterations in the lung epithelial cells. Cancer initiation is a rapid process compared with the promotion and progression phases (Figure 2). Lung cell microenvironment is changed as a result of frequent exposure to carcinogens. Carcinogens form inflammatory, reactive electrophilic metabolites and oxidative stress (reactive oxygen and nitrogen species (ROS, RNS)), which have the ability to interact with DNA and cause DNA damage [8]. Ionizing radiation can produce reactive oxygen intermediates, causing oxidative DNA damage and double strand break [76]. Polycyclic aromatic hydrocarbons, present in tobacco smoke, diesel exhaust, and soot, form DNA adducts and oxidative DNA damage leading to somatic mutation. Persistent DNA damage can cause miscoding during replication and loss of normal cell functions resulting in uncontrolled cell growth and proliferation. Genomic instability, a hallmark of cancer, is the main reason for sustained cell proliferation signals, cell death resistance and suppression invasion [42]. Prevention of genotoxicity and maintenance of genome stability at the early phase of cancer development is the most effective method of lung cancer prevention. Genome stability can be achieved by balancing oxidative stress through scavenging free radicals and/or by inducing the activity of phase II detoxifying enzymes that can detoxify and excrete carcinogenic metabolites from the body. A brief description of lung carcinogenesis is demonstrated in Figure 2.

## 5. Evidences for Lung Cancer Prevention by Dietary Polyphenols

**Figure 3:**
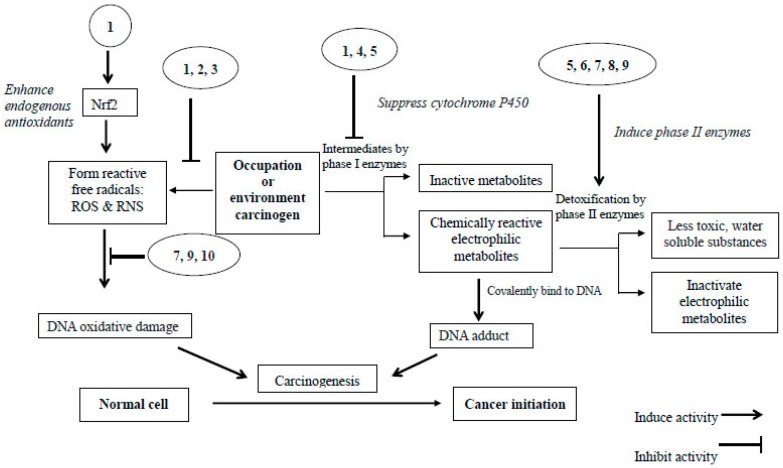
Intervention of polyphenols at lung cancer initiation stage [78,79,80]. **1**, Quercetin; **2**, Naringin; **3**, Luteolin; **4**, Keampferol; **5**, Proanthocyanidine; **6**, Baicalein; **7**, Catechins; **8**, Isothiocyanate; **9**, Epigallocatechin gallate; and **10**, Rutin.

Three chemopreventive strategies have been suggested for polyphenols by Soria et al. [77]. They are: (1) primary prevention, the prevention of cancer in healthy high risk individuals; (2) secondary prevention, preventing cancer development in individuals who are having precancerous lesions; and (3) tertiary prevention, the prevention of recurrence or metastasis in individuals who have experienced cancer before. The prevention of primary lung cancer with polyphenols has been summarized in Figure 3. 

### 5.1. In Vitro Studies

Cancer preventive properties of phytochemicals have been studied extensively; however, very little in relation to lung carcinogens. Epigallocatechin-3-gallate (EGCG), a known polyphenol present in green tea, dose dependently suppresses the hexavalent chromium (Cr (VI))-induced apoptosis, reduces activation of caspase-3 and nuclear poly (ADP-ribose) polymerase (PARP), and intracellular ROS and DNA-protein cross links in BEAS-2B cells [81]. It has been demonstrated that bisdemethoxycurcumin (curcuminoid separated from Curcuma longa (turmeric)), can prevent the premature senescence of WI-38 normal lung fibroblast cells treated with *tert*-butyl hydroperoxide (*t*-BHP), through surtuins 1/AMP-activated protein kinase (Sirt1/AMPK) signaling pathway [82]. Caffeic acid (3,4-dihydroxy cinnamic acid) can scavenge intracellular ROS and 1,1-diphenyl-2-picrylhydrazyl radical and hence prevent lipid peroxidation in WI-38 cells exposed to hydrogen peroxide (H_2_O_2_). It could increase the activity of catalase and protein expression and activate the extracellular signal regulated kinase protein and protect cells from H_2_O_2_ damage [83]. Methoxylated flavonoids and resveratrol (found abundantly in grapes) block the DNA binding of BaP, a polycyclic aromatic hydrocarbon present in tobacco smoke, and CYP1A1 protein activation in BEAS-2B normal bronchial epithelial cells [84]. Quercetin has induced Nrf2-driven heme oxygenase 1 (HO-1) expression system and its antioxidative properties respond to the changes in the cellular redox environment in BEAS-2B cells [85]. Luteolin, a flavone, is rich in kiwi fruit and melons. Tan and his colleagues have explored the chemopreventive ability of luteolin in cigarette smoke extract (CSE)-induced normal human bronchial epithelial cells (NHBE). They found that luteolin could attenuate CSE-induced apoptosis, noticeably reduce CSE-induced expression of Nrf2, nicotinamide adenine dinucleotide phosphate (NAD(P)H):Quinone oxidoreductase 1 (NQO1) and HO-1. Further, cellular glutathione (GSH) level was increased with luteolin, which triggered suppression of the ROS generation [86]. Quercetin, a common flavonol present in apples and onions, has suppressed the expression of CYP1A1 and CYP1B1 phase I enzymes in BaP treated BEAS-2B cells. Kaempferol, another member of flavonol, suppresses phase I enzymes activated by cigarette smoke condensate (CSC) in BEAS-2B cells. Further, Kaempferol could prevent the CSC-induced cell transformation and colony formation in BEAS-2B cells [84,87]. 

### 5.2. In Vivo Studies

Polyphenol studies of lung cancer prevention in experimental mice models are presented in Table 3. The chemopreventive properties of mangiferin (*C*-glycosylxanthone structure) have been shown against BaP-induced lung carcinogenesis in male Swiss albino mice, possibly through enhancing phase II enzymes [88]. 

## 6. Epidemiological Evidence of Lung Cancer Prevention

Quitting smoking and eating healthy diets are the most important healthy habits to reduce lung cancer incidence. Many studies have confirmed the reduction of lung cancer risk among people consuming fruit- and vegetable-rich diets [99]. Zhong and colleagues (2001) have found a 35% reduction of lung cancer risk in non-smoking women who regularly drink green tea compared with women who did not drink tea regularly in Shanghi, China [100]. In a health professionals’ follow-up study, over 77,200 women and 47,700 men were examined for an association between lung cancer risk and fruits and vegetables consumption. A 21% reduction in lung cancer risk was found in women with the highest fruits and vegetable consumption [101]. A similar risk reduction was not found in the men. A case-control study was conducted in Poland with 118 women diagnosed with lung cancer and 141 healthy women. It showed that cigarette smoking and drinking vodka increased lung cancer risk while frequent consumption of carrots reduced the risk [102]. However, a pooled analysis of seven cohort studies done in North America and Europe failed to show any association between β-carotene intake and lung cancer risk,. But an inverse association was found with β-cryptoxanthin from citrus fruits and lung cancer risk. A study in Singapore has further confirmed the chemopreventive effect of β-cryptoxanthin in reducing lung cancer risk [103,104]. Moreover, a population-based, case-control study in Hawaii has found a significant inverse association between lung cancer risk and regular consumption of onions, apple (main dietary source of flavonoid quercetin) and white grape fruit (naringin). The effect of onions was particularly strong against squamous cell carcinoma and it suggests that decreased activation of PAHs and other carcinogens by inhibiting the activity of cytochrome 450 enzymes. Inhibition of the activation of procarcinogens through cytochrome enzymes may be a major mechanism of polyphenols in lung cancer prevention.

A cohort study (consisting of 521,468 subjects; both men and women between 25–70 years old) conducted in 10 European countries showed an inverse association of lung cancer risk in relation to the varied consumption of fruits and vegetables among smokers. The risk of developing squamous cell carcinoma was reduced among smokers consuming a variety of fruits and/or vegetables, but no association was observed in adenocarcinomas and small cell carcinoma. Lung cancer risk is lower with increasing consumption of a variety of fruits and vegetables, independent from quantity of consumption [105]. Once the variety of fruits and vegetables in the diet is higher, diversity of bioactive compounds also increases. A greater variety of fruits and vegetable consumption therefore represents a more diverse bioactive phytochemical intake. Contrast to this finding, Linseisen et al., [7] found only fruit (apples and pears) consumption has an inverse relationship with lung cancer risk, not vegetables. However, lung cancer risk was significantly decreased in smokers who consume more vegetables, specifically root vegetables. Lycopene, β-cryptoxanthin, total carotenoids and lutein significantly reduced lung cancer risk among male smokers. In addition, lycopene reduced the risk of small cell and squamous cell carcinoma, but not adenocarcinoma [106].

## 7. Conclusions

Genome instability is the primary cause of lung cancer initiation. Almost all lung carcinogens are able to alter the cell microenvironment, which favors DNA damage. Oxidative DNA damage is the central process causing lung carcinogenesis. Plant polyphenols have exhibited multiple modes of cancer prevention functions: (i) polyphenols act as antioxidants and regulate oxidative stress caused by the lung carcinogens; hence, they mediate oxidative DNA damage, lipid autoxidation, and cell membrane damage; (ii) polyphenols inhibit the activation of cytochrome 450 enzymes (phase I enzymes), which form reactive electrophilic metabolites from pro-carcinogens. These reactive intermediary metabolites can covalently bind with DNA specific sites and form DNA adducts which activate oncogenes and suppress tumor suppressor genes, leading to lung cancer; and (iii) polyphenols enhance the activity of phase II detoxification enzymes, which are able to detoxify electrophilic metabolites from phase I enzymes and excrete them through urine and bile. Furthermore, many polyphenols enhance the activity of enzymatic (SOD, CAT, GPx) and non-enzymatic antioxidants (Vitamin E, C, and GST) that are able to balance the ROS and RNS in cells. Polyphenols can be found ubiquitously in fruits, vegetables, grains, and other plant-based food. Further investigations are required to identify specific polyphenols and their dietary sources that are involved in oxidative protection, regulation of phase I and II enzymes, and regulation of cell survival pathways in relation to lung carcinogenesis. Even though there is some controversy in reported literature, this review recognizes many in vitro, in vivo, and epidemiological studies supporting the notion that habitual dietary intervention of polyphenols can reduce the risk of lung cancer.

## Figures and Tables

**Table 1:** In vitro studies of anti-proliferative effect of polyphenols in lung cancer.

Cell Type	Polyphenol	Proposed Mechanism of Action	Reference
A549, H460 & H1299	Grape seed proanthocyanidins	Inhibit cell migration and endogenous nitric oxide Inhibit activation of ERK1/2 Induce apoptosis Activate caspases-9 and -3 Activate poly (ADP-ribose) polymerase	[35,36,37]
NCI-H209	Quercetin glucuronides	Decrease cell viability (dose and time dependent) Arrest cell cycle at G2/M phase via caspase-3 cascade	[38]
A549 & H460	Curcumin	Inhibit cell proliferation Induce fork head box protein O1 (FOXO1) expression	[39]
PC-9	Curcumin	Inhibit cell growth Induce G1/S arrest via activating CDK inhibitor genes *p21* and *p27*	[40]
A549	Polyphenol rich brown alga (*Ecklonia cava*) extract	Suppress migration and invasion Down-regulate MMP-2 activity Anti-metastatic effect	[41]

Non-small cell lung cancer (NSCLC) cell—A549, H460, H1299; human adenocarcinoma cell—PC-9; small cell lung cancer (SCLC) cell—NCI-H209; extracellular signal-regulated kinase—ERK; adenosine diphosphate—ADP; cyclin dependent kinase—CDK; and matrix metalloproteinase—MMP.

**Table 2:** Group I lung carcinogens classified by International Agency for Research on Cancer (IARC) (2012).

Group 1 Carcinogens	Type of Exposure
**Personal Habits and Indoor Combustion**	
Tobacco Smoking and Second Hand Smoke	E
Household Combustion of Coal Tar	E
Diesel Exhaust	E, O
**Chemical Agents and Related Occupation**	
benzo[a]pyrene (BaP)	O
Coal Gasification	O
Coal-tar Pitch	O
Coke Production	O
Soot (Contains BaP)	E, O
Aluminium Production	O
Bis(chloromethyl)ether and Chloromethyl Methyl Ether	O
Sulfur Mustard	O
Iron and steel founding	O
Painting	O
Rubber Manufacturing	O
**Radiation**	
X-radiation and γ-radiation	O
Internalized α-particle Emitting Radionuclides Radon (Rn)—^222^Rn Produced from Uranium (^238^U) and ^220^Rn Produced from Thorium Plutonium-239	E, O
**Metal, Fiber and Dust**	
Arsenic and Inorganic Arsenic Compounds	E, O
Beryllium and its Compounds	E, O
Cadmium and Cadmium Compounds	E, O
Chromium(VI) Compounds	E, O
Nickel compounds	E, O
Asbestos	E, O
Crystalline silica in the form of quartz or cristobalite	E, O
**Pharmaceuticals**	
Mechlorethamine, Oncovin, Procarbazine, and Prednisone (MOPP) combination therapy	O

Second-hand smoke—side stream smoke emitted into the environment from the smoldering of cigarettes and other tobacco products between puffs and from the mainstream smoke exhaled by the smoker; environment—E; occupation—O [50,51,52,53,54,55,56,57,58,59,60,61,62,63,64,65,66,67,68,69,70,71,72,73,74].

**Table 3:** Polyphenols in cancer prevention: in vivo studies.

Animal Model	Carcinogen	Compound or Extract	Observation	Reference
Swiss ICR Mice	Cigarette smoke (CS)	Black chokeberry and strawberry aqueous extracts	Reduce cytogenetic damage, liver degeneration, pulmonary emphysema and lung adenomas Inhibit CS-related body weight loss	[89]
Mice	BaP	Hesperidin	Attenuate mast cell density Down regulate expressions of COX-2, MMP-2 and MMP-9 Exert anti-carcinogenic activity against lung cancer	[90]
Swiss Albino Mice	BaP	Baicalein	Increase enzyme antioxidants and non-enzyme antioxidants Decrease the activity of phase I enzymes Increase the activity of phase II detoxification enzymes Preserve pulmonary microvasculature and normal growth pattern	[91]
Swiss Albino mice	BaP	Mangiferin	Prevent decrement of electron transport chain complexes and TCA cycle key enzymes in lung cancer bearing mice	[92]
ICR Mice	Tobacco smoke	Apple polyphenol	Reduced inflammation Reverse oxidative stress in lung tissues Regulate the MMP-9 in cells	[93]
Swiss Albino Mice	BaP	Naringenin	Activate the enzymatic antioxidants (SOD, CAT, GPx, GST) Suppress unregulated expression of CYP1A1, PCNA and NF-κB Reduce pro-inflammatory cytokines (TNF-α, IL-6 and IL-1β) Reduce proliferative lesions in lung	[94]
Swiss Albino Mice	BaP	Fisetin	Restore lipid peroxidase, enzymatic and non-enzymatic antioxidants levels Reduce the lung lesions Reduce PCNA	[95]
A/J Mice	NNK	EGCG	Attenuate the induction of DNMT1 Reduce phospho-histone H2AX (γ-H2AX) and phospho-AKT (p-AKT)	[96]
Sprague-Dawley Rats	NNK	Cape gooseberry extract	Reduce pulmonary hyperplasia Improve the DNA content Reduce expression of cell proliferation marker Ki-67 Enhance expression of tumor suppressor gene *p53*	[97]
Mongolian Gerbils	BaP	Quercetin	Suppress the expression of TNF-α, IL-1β, phospho-c-Jun and phospho-JNK	[98]

Cyclooxygenase-2—COX-2; matrix metalloprotein—MMP; tricarboxylic acid—TCA; superoxide dismutase—SOD; catalase—CAT; glutathione peroxidase—GPx; Glutathione—GST; proliferating cell nuclear antigen—PCNA; nuclear factor-κ light-chain-enhancer of activated B cells—NF-κB; Tumor necrosis factor-α—TNF-α; Interleukin—IL; 4-(methylnitro-samino)-1-(3-pyridyl)-1-butanone—NNK; and DNA methyltransferase 1—DNMT 1.
